# Obituaries

**Published:** 1913-02

**Authors:** 


The editor has been appointed to the Committee on Necrology of the Medical Society of the County of Erie, N. Y. It is understood that the appointment is not an honor but an assignment to service such as every member is glad to perform as a matter of public spirit. As the BUFFALO MEDICAL JOURNAL is trying to publish all changes in the personel of the profession of Western New York, this duty can be discharged without much additional effort, but, obviously, the necessary information must be supplied by immediate friends and members of families. The editor frankly admits his inability to write the eulogistic and religious matter so often injected into obituaries, and wishes to state in advance that the general limitation of obituaries to biographic, genealogic and statistic details in as condensed form as possible, will not indicate a lack of proper feeling nor a failure to appreciate the worth of the deceased.

